# Supplementary material for: Pre-Emptive Drug Safety Evaluation of Iclepertin (BI-425809) Using Real-World Data and Virtual Addition of This Medication to the Actual Drug Regimen of Individuals from Large Populations
Source: Pharmaceuticals (Basel). 2025 Sep 28;18(10):1453. doi: 10.3390/ph18101453 (PMC12566928; doi:10.3390/ph18101453)
Supplement: Supplementary file 1 [file pharmaceuticals-18-01453-s001.zip › pharmaceuticals-3875849-supplementary.pdf]

pharmaceuticals

MDPI

Supplementary Table S1. The top 50 prescribed medications by coverage population.

Commercial group (n=1,937,389)		Medicaid group (n =1,983,976)		Medicare group (n=483,698)	
Drug name	Number of individuals, n (%)	Drug name	Number of individuals, n (%)	Drug name	Number of individuals, n (%)
Hydrochlorothiazide	178,881 (9.2%)	Acetaminophen	206,668 (10.4%)	Atorvastatin	94,774 (19.6%)
Lisinopril	173,088 (8.9%)	Lisinopril	201,929 (10.2%)	Hydrochlorothiazide	72,546 (15.07%)
Ethinyl estradiol	172,760 (8.9%)	Albuterol	196,194 (9.9%)	Thyroxine	71,923 (14.9%)
Atorvastatin	162,952 (8.4%)	Atorvastatin	192,944 (9.7%)	Lisinopril	70,733 (14.6%)
Thyroxine	153,899 (7.9%)	Gabapentin	187,612 (9.5%)	Amlodipine	69,073 (14.3%)
Amoxicillin	140,015 (7.2%)	Ibuprofen	172,234 (8.7%)	Metoprolol	67,893 (14.0%)
Metformin	128,646 (6.6%)	Omeprazole	163,914 (8.3%)	Losartan	54,704 (11.3%)
Amlodipine	122,391 (6.3%)	Fluticasone	159,241 (8.0%)	Metformin	46,821 (9.7%)
Losartan	101,569 (5.2%)	Amlodipine	148,863 (7.5%)	Simvastatin	42,569 (8.8%)
Acetaminophen	96,596 (5.0%)	Hydrochlorothiazide	145,183 (7.3%)	Omeprazole	35,377 (7.3%)
Fluticasone	92,929 (4.8%)	Metformin	144,797 (7.3%)	Rosuvastatin	28,450 (5.9%)
Metoprolol	83,070 (4.3%)	Thyroxine	122,313 (6.2%)	Tamsulosin	28,112 (5.8%)
Norethindrone	82,238 (4.2%)	Metoprolol	117,796 (5.9%)	Furosemide	25,884 (5.4%)
Omeprazole	80,118 (4.1%)	Ethinyl estradiol	111,939 (5.6%)	Fluticasone	24,928 (5.2%)
Azithromycin	78,842 (4.1%)	Amoxicillin	107,606 (5.4%)	Gabapentin	22,932 (4.7%)
Ibuprofen	77,309 (4.0%)	Trazodone	100,606 (5.4%)	Carvedilol	21,181 (4.2%)
Albuterol	77,236 (4.0%)	Sertraline	98,153 (4.9%)	Potassium chloride	19,826 (4.1%)
Prednisone	74,495 (3.8%)	Oxycodone	96,537 (4.9%)	Pravastatin	19,826 (4.1%)
Sertraline	70,783 (3.7%)	Hydrocodone	93,946 (4.7%)	Pantoprazole	19,316 (4.0%)
Escitalopram	68,097 (3.5%)	Cetirizine	88,444 (4.5%)	Apixaban	18,075 (3.7%)
Bupropion	65,807 (3.4%)	Acetylsalicylic acid	86,680 (4.4%)	Clopidogrel	17,457 (3.6%)
Clavulanic acid	64,535 (3.3%)	Loratadine	83,467 (4.2%)	Acetaminophen	16,598 (3.4%)
Montelukast	64,415 (3.3%)	Ergocalciferol	83,008 (4.2%)	Atenolol	15,651 (3.2%)
Hydrocodone	61,163 (3.2%)	Pantoprazole	81,414 (4.1%)	Sertraline	14,683 (3.0%)
Rosuvastatin	59,980 (3.1%)	Cholecalciferol	80,902 (4.1%)	Allopurinol	14,470 (3.0%)
Meloxicam	50,829 (2.6%)	Prednisone	75,612 (3.8%)	Montelukast	14,144 (2.9%)
Gabapentin	50,550 (2.6%)	Cyclobenzaprine	75,078 (3.8%)	Latanoprost	13,291 (2.7%)
Ferrous fumarate	48,657 (2.5%)	Hydroxyzine	74,497 (3.8%)	Albuterol	13,107 (2.7%)
Simvastatin	48,431 (2.5%)	Losartan	73,841 (3.7%)	Amoxicillin	13,037 (2.7%)
Pantoprazole	48,227 (2.5%)	Bupropion	73,659 (3.7%)	Prednisone	11,994 (2.5%)
Doxycycline	45,732 (2.4%)	Furosemide	70,593 (3.6%)	Finasteride	11,586 (2.4%)
Cyclobenzaprine	44,154 (2.3%)	Montelukast	68,185 (3.4%)	Trazodone	11,292 (2.3%)
Estradiol	43,292 (2.2%)	Ondansetron	66,811 (3.4%)	Hydrocodone	11,199 (2.3%)
Fluoxetine	43,068 (2.2%)	Ranitidine	62,774 (3.2%)	Escitalopram	11,170 (2.3%)
Ergocalciferol	42,846 (2.2%)	Meloxicam	62,087 (3.1%)	Diltiazem	11,016 (2.3%)

Benzonatate	41,640 (2.1%)	Duloxetine	61,883 (3.1%)	Meloxicam	11,006 (2.3%)
Methylprednisolone	41,419 (2.1%)	Fluoxetine	61,378 (3.1%)	Varicella zoster vaccine	10,304 (2.1%)
Dextroamphetamine	41,329 (2.1%)	Quetiapine	60,240 (3.0%)	Warfarin	10,083 (2.1%)
Norgestimate	41,276 (2.1%)	Potassium chloride	59,536 (3.0%)	Alendronate	9,994 (2.1%)
Amphetamine	40,888 (2.1%)	Escitalopram	58,948 (3.0%)	Ezetimibe	9,982 (2.1%)
Valacyclovir	39,740 (2.1%)	Naproxen	58,520 (2.9%)	Valsartan	9,581 (2.0%)
Alprazolam	37,655 (1.9%)	Buspirone	56,520 (2.8%)	Timolol	9,514 (2.0%)
Trazodone	36,115 (1.9%)	Docusate	56,328 (2.8%)	Spironolactone	9,514 (2.0%)
Citalopram	35,700 (1.8%)	Azithromycin	54,526 (2.7%)	Citalopram	9,429 (1.9%)
Ondansetron	35,203 (1.8%)	Metronidazole	54,253 (2.7%)	Glipizide	9,087 (1.9%)
Oxycodone	32,851 (1.7%)	Citalopram	52,247 (2.6%)	Rivaroxaban	8,958 (1.9%)
Trimethoprim	31,440 (1.6%)	Norethindrone	51,160 (2.6%)	Tramadol	8,866 (1.8%)
Naproxen	30,870 (1.6%)	Carvedilol	50,559 (2.5%)	Insulin glargine	8,826 (1.8%)
Duloxetine	30,689 (1.6%)	Insulin glargine	49,869 (2.5%)	Benazepril	8,773 (1.8%)
Cephalexin	30,587 (1.6%)	Famotidine	47,924 (2.4%)	Duloxetine	8,702 (1.8%)

Supplementary Table S2. Clinically relevant concomitant CYP3A4 interacting drugs including CYP3A4 inhibitors, CYP3A4 inducers, and potential interacting CYP3A4 competitive substrates (excluding weak affinity substrates).*

**Commercial group**			**Medicaid group**			**Medicare group**		
CYP3A4 drug name	**Number of Individuals, n (%)**		CYP3A4 drug name	**Number of Individuals, n (%)**		CYP3A4 drug name	**Number of Individuals, n (%)**	
	N	%		N	%		N	%
Atorvastatin	162,952	8.41	Atorvastatin	192,944	9.73	Atorvastatin	94,774	19.57
Amlodipine	122,391	6.32	Omeprazole	163,914	8.26	Amlodipine	69,073	14.26
Omeprazole	80,118	4.14	Amlodipine	148,863	7.50	Simvastatin	42,569	8.79
Simvastatin	48,431	2.50	Buspirone	56,459	2.85	Omeprazole	35,377	7.30
Doxycycline	45,732	2.36	Topiramate	45,552	2.30	**Diltiazem**	11,016	2.27
**Fluconazole**	29,896	1.54	Buprenorphine	42,816	2.16	**Warfarin**	10,083	2.08
Topiramate	19,911	1.03	**Fluconazole**	42,235	2.13	Doxycycline	6,439	1.33
Buspirone	18,736	0.97	Simvastatin	41,542	2.09	Lovastatin	6,267	1.29
Esomeprazole	13,263	0.68	Doxycycline	37,354	1.88	Esomeprazole	5,283	1.09
**Diltiazem**	10,228	0.53	Risperidone	26,572	1.34	Nifedipine	4,333	0.89
Pioglitazone	8,014	0.41	Esomeprazole	15,716	0.79	Pioglitazone	4,083	0.84
Nifedipine	7,450	0.38	**Diltiazem**	14,002	0.71	**Amiodarone**	3,693	0.76
Lovastatin	6,215	0.32	**Warfarin**	13,435	0.68	**Verapamil**	3,545	0.73
Butalbital	6,087	0.31	Nifedipine	12,482	0.63	Buspirone	3,217	0.66
**Warfarin**	5,899	0.30	Carbamazepine	10,618	0.54	Topiramate	1,588	0.33
**Verapamil**	5,654	0.29	Butalbital	9,813	0.49	**Fluconazole**	1,457	0.30
Buprenorphine	4,566	0.24	Lovastatin	8,999	0.45	Primidone	1,410	0.29
Tamoxifen	4,038	0.21	Pioglitazone	7,654	0.39	Ticagrelor	1,172	0.24
**Clarithromycin**	3,497	0.18	Phenytoin	6,753	0.34	**Ranolazine**	1,168	0.24
**Methimazole**	3,259	0.17	**Verapamil**	6,047	0.30	Felodipine	1,018	0.21

Ticagrelor	3,052	0.16	Loperamide	4,632	0.23	Risperidone	942	0.19
Carbamazepine	2,423	0.13	**Cobicistat**	4,328	0.22	Cilostazol	819	0.17
Modafinil	2,369	0.12	**Amiodarone**	3,970	0.20	Carbamazepine	786	0.16
Risperidone	2,338	0.12	**Methimazole**	3,920	0.20	**Methimazole**	777	0.16
Eletriptan	2,055	0.11	Ticagrelor	3,515	0.18	Tamoxifen	720	0.15
**Cobicistat**	1,880	0.10	**Ranolazine**	3,497	0.18	Dronedarone	664	0.14
Armodafinil	1,470	0.08	Primidone	2,508	0.13	Phenytoin	655	0.14
**Amiodarone**	1,282	0.07	Cilostazol	2,466	0.12	Butalbital	476	0.10
**Orphenadrine**	1,256	0.06	Phenobarbital	2,451	0.12	Eplerenone	475	0.10
Hydrocortisone	1,144	0.06	**Darunavir**	2,384	0.12	Repaglinide	457	0.09
**Ranolazine**	1,033	0.05	**Clarithromycin**	2,213	0.11	Bicalutamide	427	0.09
Phenytoin	994	0.05	Tamoxifen	2,177	0.11	Hydrocortisone	416	0.09
			**Orphenadrine**	1,974	0.10	Modafinil	352	0.07
			Hydrocortisone	1,890	0.10	Loperamide	340	0.07
			Clobazam	1,887	0.10	**Clarithromycin**	254	0.05
			Modafinil	1,314	0.07	Phenobarbital	231	0.05
			Quinidine	1,085	0.05	Abiraterone	219	0.05

* CYP3A4 inhibitors, substrate-inhibitors, and strong affinity substrates (competitive inhibitors) are shown in bold.

References

1. Le Louët, H., and P. J. Pitts. "Twenty-First Century Global Adr Management: A Need for Clarification, Redesign, and Coordinated Action." *Ther Innov Regul Sci* 57, no. 1 (2023): 100-03.
2. American Society of Pharmacovigilance. "America's Silent Epidemic Worsens: Adverse Drug Events Now the Third Leading Cause of Death." PR Newswire.
3. Bonn, D. "Adverse Drug Reactions Remain a Major Cause of Death." *Lancet* 351, no. 9110 (1998): 1183.
4. Bates, D. W. "Drugs and Adverse Drug Reactions: How Worried Should We Be?" *Jama* 279, no. 15 (1998): 1216-7.
5. Lavertu, A., B. Vora, K. M. Giacomini, R. Altman, and S. Rensi. "A New Era in Pharmacovigilance: Toward Real-World Data and Digital Monitoring." *Clin Pharmacol Ther* 109, no. 5 (2021): 1197-202.
6. Giacomini, K. M., R. M. Krauss, D. M. Roden, M. Eichelbaum, M. R. Hayden, and Y. Nakamura. "When Good Drugs Go Bad." *Nature* 446, no. 7139 (2007): 975-7.
7. Vargesson, N. "Thalidomide-Induced Teratogenesis: History and Mechanisms." *Birth Defects Res C Embryo Today* 105, no. 2 (2015): 140-56.
8. Wang, C. W., I. A. C. Preclaro, W. H. Lin, and W. H. Chung. "An Updated Review of Genetic Associations with Severe Adverse Drug Reactions: Translation and Implementation of Pharmacogenomic Testing in Clinical Practice." *Front Pharmacol* 13 (2022): 886377.
9. Wu, Z., P. Zhou, N. He, and S. Zhai. "Drug-Induced Torsades De Pointes: Disproportionality Analysis of the United States Food and Drug Administration Adverse Event Reporting System." *Front Cardiovasc Med* 9 (2022): 966331.
10. Pinnow, E., S. Amr, S. M. Bentzen, S. Brajovic, L. Hungerford, D. M. St George, and G. Dal Pan. "Postmarket Safety Outcomes for New Molecular Entity (Nme) Drugs Approved by the Food and Drug Administration between 2002 and 2014." *Clin Pharmacol Ther* 104, no. 2 (2018): 390-400.
11. Lester, J., G. A. Neyarapally, E. Lipowski, C. F. Graham, M. Hall, and G. Dal Pan. "Evaluation of Fda Safety-Related Drug Label Changes in 2010." *Pharmacoepidemiol Drug Saf* 22, no. 3 (2013): 302-5.
12. Silva-Almodóvar, Armando, and Milap C. Nahata. "Clinical Utility of Medication-Based Risk Scores to Reduce Polypharmacy and Potentially Avoidable Healthcare Utilization." *Pharmaceuticals* 15, no. 6 (2022): 681.
13. Organization, World Health. "Multimorbidity." In *Multimorbidity*, 2016.
14. Wouters, H., H. van der Meer, and K. Taxis. "Quantification of Anticholinergic and Sedative Drug Load with the Drug Burden Index: A Review of Outcomes and Methodological Quality of Studies." *Eur J Clin Pharmacol* 73, no. 3 (2017): 257-66.
15. Taipale, H. T., J. S. Bell, D. Gnjidic, R. Sulkava, and S. Hartikainen. "Sedative Load among Community-Dwelling People Aged 75 Years or Older: Association with Balance and Mobility." *J Clin Psychopharmacol* 32, no. 2 (2012): 218-24.
16. Turgeon, J., and V. Michaud. "Clinical Decision Support Systems: Great Promises for Better Management of Patients' Drug Therapy." *Expert Opin Drug Metab Toxicol* 12, no. 9 (2016): 993-5.
17. Doan, J., H. Zakrzewski-Jakubiak, J. Roy, J. Turgeon, and C. Tannenbaum. "Prevalence and Risk of Potential Cytochrome P450-Mediated Drug-Drug Interactions in Older Hospitalized Patients with Polypharmacy." *Ann Pharmacother* 47, no. 3 (2013): 324-32.
18. Michaud, V., M. K. Smith, R. Bikmetov, P. Dow, J. Johnson, A. Stein, S. Finnel, H. Jin, and J. Turgeon. "Association of the Medwise Risk Score with Health Care Outcomes." *Am J Manag Care* 27, no. 16 Suppl (2021): S280-s91.
19. Ratigan, A. R., V. Michaud, J. Turgeon, R. Bikmetov, G. Gaona Villarreal, H. D. Anderson, G. Pulver, and W. D. Pace. "Longitudinal Association of a Medication Risk Score with Mortality among Ambulatory Patients Acquired through Electronic Health Record Data." *J Patient Saf* 17, no. 4 (2021): 249-55.
20. Zakrzewski-Jakubiak, H., J. Doan, P. Lamoureux, D. Singh, J. Turgeon, and C. Tannenbaum. "Detection and Prevention of Drug-Drug Interactions in the Hospitalized Elderly: Utility of New Cytochrome P450-Based Software." *Am J Geriatr Pharmacother* 9, no. 6 (2011): 461-70.

21. Bankes, D., K. Pizzolato, S. Finnel, M. S. Awadalla, A. Stein, J. Johnson, and J. Turgeon. "Medication-Related Problems Identified by Pharmacists in an Enhanced Medication Therapy Management Model." *Am J Manag Care* 27, no. 16 Suppl (2021): S292-s99.
22. Stein, A., S. Finnel, D. Bankes, H. Jin, M. S. Awadalla, J. Johnson, and J. Turgeon. "Health Outcomes from an Innovative Enhanced Medication Therapy Management Model." *Am J Manag Care* 27, no. 16 Suppl (2021): S300-s08.
23. European Centre for Disease Prevention and Control. "Cluster of Pneumonia Cases Caused by a Novel Coronavirus, Wuhan, China, 2020." https://www.ecdc.europa.eu/en/publications-data/rapid-risk-assessment-cluster-pneumonia-cases-caused-novel-coronavirus-wuhan (accessed 8 January 2023).
24. World Health Organization. "Pneumonia of Unknown Cause – China." https://www.who.int/emergencies/disease-outbreak-news/item/2020-DON229 (accessed 8 January 2023).
25. Govender, K., and A. Chuturgoon. "An Overview of Repurposed Drugs for Potential Covid-19 Treatment." *Antibiotics (Basel)* 11, no. 12 (2022).
26. Michaud, V., P. Dow, S. B. Al Rihani, M. Deodhar, M. Arwood, B. Cicali, and J. Turgeon. "Risk Assessment of Drug-Induced Long Qt Syndrome for Some Covid-19 Repurposed Drugs." *Clin Transl Sci* 14, no. 1 (2021): 20-28.
27. Al Rihani, S. B., M. K. Smith, R. Bikmetov, M. Deodhar, P. Dow, J. Turgeon, and V. Michaud. "Risk of Adverse Drug Events Following the Virtual Addition of Covid-19 Repurposed Drugs to Drug Regimens of Frail Older Adults with Polypharmacy." *J Clin Med* 9, no. 8 (2020).
28. Smith, M. K., R. Bikmetov, S. B. Al Rihani, M. Deodhar, M. Hafermann, P. Dow, J. Turgeon, and V. Michaud. "Adverse Drug Event Risk Assessment by the Virtual Addition of Covid-19 Repurposed Drugs to Medicare and Commercially Insured Patients' Drug Regimens: A Drug Safety Simulation Study." *Clin Transl Sci* 14, no. 5 (2021): 1799-809.
29. Bankes, D. L., H. Jin, S. Finnel, V. Michaud, C. H. Knowlton, J. Turgeon, and A. Stein. "Association of a Novel Medication Risk Score with Adverse Drug Events and Other Pertinent Outcomes among Participants of the Programs of All-Inclusive Care for the Elderly." *Pharmacy (Basel)* 8, no. 2 (2020).
30. Deodhar, M., S. B. Al Rihani, M. J. Arwood, L. Darakjian, P. Dow, J. Turgeon, and V. Michaud. "Mechanisms of Cyp450 Inhibition: Understanding Drug-Drug Interactions Due to Mechanism-Based Inhibition in Clinical Practice." *Pharmaceutics* 12, no. 9 (2020).
31. Hreiche, Raymond, Pierre Morissette, and Jacques Turgeon. "Drug-Induced Long Qt Syndrome in Women: Review of Current Evidence and Remaining Gaps." *Gender Medicine* 5, no. 2 (2008): 124-35.
32. Makkar, R. R., B. S. Fromm, R. T. Steinman, M. D. Meissner, and M. H. Lehmann. "Female Gender as a Risk Factor for Torsades De Pointes Associated with Cardiovascular Drugs." *Jama* 270, no. 21 (1993): 2590-7.
33. Rosenbrock, H., M. Desch, and G. Wunderlich. "Development of the Novel Glyt1 Inhibitor, Iclepertin (Bi 425809), for the Treatment of Cognitive Impairment Associated with Schizophrenia." *Eur Arch Psychiatry Clin Neurosci* 273, no. 7 (2023): 1557-66.
34. Rast, G., and B. D. Guth. "Solubility Assessment and on-Line Exposure Confirmation in a Patch-Clamp Assay for Herg (Human Ether-a-Go-Go-Related Gene) Potassium Channel Inhibition." *J Pharmacol Toxicol Methods* 70, no. 2 (2014): 182-7.
35. Cicali, Brian; Michaud, Veronique; Knowlton, Calvin; Turgeon Jacques;. "Application of a Novel Medication-Related Risk Stratification Strategy to a Self-Funded Employer Population." *Benefits Quarterly* 34, no. 2 (2018): 49-55.
36. Turgeon, J, Stefen LE, Badea G, Michaud V. "Treatment Methods Having Reduced Drug-Related Toxicity and Methods of Identifying the Likelihood of Patient Harm for Prescribed Medications." In *U.S. Patent and Trademark Office*. United States, 2021.
37. Charlson, M. E., P. Pompei, K. L. Ales, and C. R. MacKenzie. "A New Method of Classifying Prognostic Comorbidity in Longitudinal Studies: Development and Validation." *J Chronic Dis* 40, no. 5 (1987): 373-83.
38. Lin, Mingfeng, Henry C. Lucas Jr, and Galit Shmueli. "Research Commentary—Too Big to Fail: Large Samples and the P-Value Problem." *Information Systems Research* 24, no. 4 (2013): 906-17.

Disclaimer/Publisher's Note: The statements, opinions and data contained in all publications are solely those of the individual author(s) and contributor(s) and not of MDPI and/or the editor(s). MDPI and/or the editor(s) disclaim responsibility for any injury to people or property resulting from any ideas, methods, instructions or products referred to in the content.
